# Effective microtissue RNA extraction coupled with Smart-seq2 for reproducible and robust spatial transcriptome analysis

**DOI:** 10.1038/s41598-020-63495-6

**Published:** 2020-04-27

**Authors:** Miki Yamazaki, Masahito Hosokawa, Koji Arikawa, Kiyofumi Takahashi, Chikako Sakanashi, Takuya Yoda, Hiroko Matsunaga, Haruko Takeyama

**Affiliations:** 1 Department of Life Science and Medical Bioscience, Waseda University, 2-2 Wakamatsu-cho, Shinjuku-ku, Tokyo, 162–8480, Japan; 2 Computational Bio Big-Data Open Innovation Laboratory, AIST-Waseda University, 3-4-1 Okubo, Shinjuku-ku, Tokyo, 169–0072, Japan; 3 Institute for Advanced Research of Biosystem Dynamics, Waseda Research Institute for Science and Engineering, Waseda University, 2-2 Wakamatsu-cho, Shinjuku-ku, Tokyo 162-8480, Japan; 4 Research Organization for Nano & Life Innovation, Waseda University, 513 Wasedatsurumaki-cho, Shinjuku-ku, Tokyo, 162–0041, Japan

**Keywords:** RNA sequencing, Transcriptomics, Gene expression profiling

## Abstract

Spatial transcriptomics is useful for understanding the molecular organization of a tissue and providing insights into cellular function in a morphological context. In order to obtain reproducible results in spatial transcriptomics, we have to maintain tissue morphology and RNA molecule stability during the image acquisition and biomolecule collection processes. Here, we developed a tissue processing method for robust and reproducible RNA-seq from tissue microdissection samples. In this method, we suppressed RNA degradation in fresh-frozen tissue specimens by dehydration fixation and effectively collected a small amount of RNA molecules from microdissection samples by magnetic beads. We demonstrated the spatial transcriptome analysis of the mouse liver and brain in serial microdissection samples (100 μm in a diameter and 10 μm in thickness) produced by a microdissection punching system. Using our method, we could prevent RNA degradation at room temperature and effectively produce a sequencing library with Smart-seq2. This resulted in reproducible sequence read mapping in exon regions and the detection of more than 2000 genes compared to non-fixed samples in the RNA-seq analysis. Our method would be applied to various transcriptome analyses, providing the information for region specific gene expression in tissue specimens.

## Introduction

Transcriptome analysis allows us to comprehensively characterise the transcripts present in biological tissues^1,2^. Conventional transcriptomes are typically performed by sequencing of RNA samples extracted from homogenized biopsies, but they result in averaged transcripts and loss of spatial information. In order to overcome these issues, current spatial transcriptome techniques have been developed to analyse spatial and specific transcript distributions in tissues. These techniques include laser capture microdissection (LCM)-based RNA-seq^3,4^, slide-based tissue-positional transcript barcoding^5–7^, and single-cell RNA-seq with computer modeling^8,9^. The spatial transcriptome analysis can provide us with insights into the molecular organization of tissues and organs and how their disorder is associated with function and disease^10,11^.

Serial microtomy sequencing analysis is a straightforward technique to obtain two-dimensional (2D) or three-dimensional (3D) transcriptional maps in complex tissues. However, in order to analyse a wide tissue area, we have to acquire tissue images and collect tissue microdissection samples in high resolution. Hence, the degradation of RNA and tissue morphological changes should be avoided to link the images and gene expression profiles. RNA degradation would induce coverage bias in transcript sequences, resulting in a disturbance in the estimation of gene expression^12,13^. Thus, the tissue preparation process should be optimized for robust and reproducible spatial transcriptomics.

So far, we have developed a semi-automated system for multiple tissue microdissection collection^14^. By using a fine stainless-steel hollow needle with a diameter of 100 μm, this system can semi-automatically capture a series of tissue microdissection samples in a short period (5 sec/sampling cycle) by punching tissue slices. The negligibly small rate of cross-contamination in transcripts has been verified from the serially collected samples and collection buffer. By combining this technique with RNA-seq, we have demonstrated a spatial transcriptome analysis of disc-shaped areas at 100 μm-diameter resolution and 20 μm in height in mouse brain tissue. Since this system allows the collection of specific tissue areas on demand, we can easily analyze the link between an anatomical region and specific gene expressions.

Here, based on our tissue microdissection collecting system, we report a tissue processing method for microtissue RNA-seq with suppressing RNA degradation of fresh-frozen tissue specimens. First, we evaluated how the RNA degradation and insufficient tissue lysis effect on the sequence read proportions, detected gene numbers, and inter-sample reproducibility in RNA-seq analysis. Then, by combining the proposed tissue fixation and RNA purification procedure with the tissue microdissection system and Smart-seq2, we demonstrated comparable sensitivity and reproducibility in gene expression analysis from microtissues to bulk RNA-seq analysis. Then, we demonstrated the tissue area-specific gene expression in mouse brain tissues with the proposed method. The results of spatial transcriptomics analysis indicated that our microtissue RNA-seq procedure could be applied for analysing various tissue samples.

## Results and discussion

### Tissue fixation for prevention of RNA degradation

**Figure 1:**
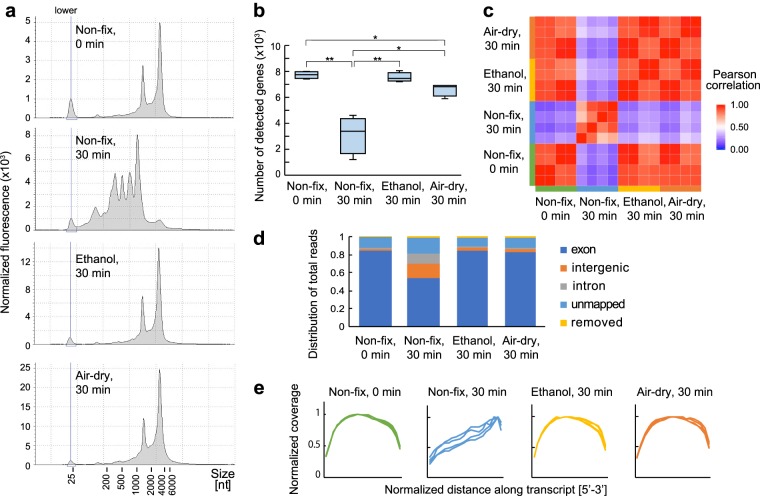
Evaluation of tissue fixation effects on RNA degradation prevention. The mouse liver tissues were treated with different fixation conditions and then total RNA was extracted from each tissue 30 min after slicing. The tissues were serially sectioned from the same mouse liver and used for RNA extraction. (**a**) Electropherograms of RNA extracted from tissue sections treated with four different conditions: no fixation 0 min and 30 min after slicing, ethanol fixation, and air-dry fixation. (**b**) The number of protein-coding genes detected from RNA-seq. The number of stars indicate p-value determined by Welch’s t-test, 1 star for p-value <0.01 and 2 stars for p-value <0.005. (**c**) Correlation heat map of gene expression levels between each sample. (**d**) Sequencing read proportions assessed by mapping to a reference genome. (**e**) Normalized average read coverage shown across the percentile predicted transcript length (5′ to 3′). Protein cording genes were used to calculate the gene coverage.

In order to optimize the tissue fixation conditions for reproducible RNA-seq from fresh-frozen microtissues, we first assessed several tissue fixation conditions in the mouse liver tissue. In general, the liver tissue shows rapid degradation compared to other organ tissues at room temperature^15^ (Fig. S1); thus, we used the liver as a model for our study. The fresh-frozen liver tissue was sectioned serially and treated with two different dehydration conditions: ethanol fixation or air-dry fixation. The electropherograms of total RNA extracted from tissue sections (Fig. 1a) showed significant degradation in the liver tissue section incubated for 30 min at room temperature after slicing. The peaks of 18 S and 28 S ribosomal RNA were clearly shifted to a shorter size in the no fixation sample. On the other hand, the liver tissue section fixed by ethanol and air-drying showed no obvious changes in electropherograms compared to the control sample. The RNA integrity number equivalent (RINe) remained at high levels (8.4 ± 0.11 and 7.9 ± 0.16, respectively). These results indicated that the RNase would decompose the RNA in tissue during incubation, but the dehydration fixation was effective to prevent RNA degradation by inactivating RNase, while stabilising the RNAs in the tissue.

Next, we compared the qualities of RNA-seq data obtained from 500 pg of total RNA extracted from each sample. The number of genes detected from the non-treated tissue decreased from 7715 ± 275 to 3132 ± 1437 genes (41%) after a 30-minute incubation at room temperature following cryosectioning (Fig. 1b). However, the fixed tissues showed statistically significant improvements in recovery rates of detectable genes, with 7536 ± 355 genes (98%) in the ethanol-fixed sample and 6607 ± 484 (86%) in the air-dry fixed sample; the coefficient of variation in detected numbers of genes decreased significantly from 0.46 in the non-treated tissue to 0.05–0.07 in the fixed tissues. With respect to the differences between the two fixation conditions, fixation of the whole tissue is considered to be non-uniform and insufficient in the air-dry fixed sample compared with ethanol fixation because a tissue section was dried gradually from the tissue surface in 5 min. With respect to the reproducibility for the assessment of gene expression patterns (Fig. 1c) in the non-fixed sample, the correlation of gene expression profiles was significantly decreased by RNA degradation over time. In contrast, the two differently fixed samples showed good correlations with the control samples, although the ethanol-fixed sample exhibited more similarity with fresh tissue samples in terms of detected gene numbers (Fig. 1b).

Moreover, we assessed the proportion of reads aligning to exonic regions, intronic regions, and intergenic regions (Fig. 1d). In the Smart-seq2 workflow, oligo(dT) was used to prime cDNA synthesis from 3ʹ poly(A) tail and full-length cDNA is amplified due to template switching. Hence, the sequence reads would have a greater fraction of reads aligning to exons. However, the non-treated sample showed a lower proportion of reads that mapped to the exon region (48–58%), while the ethanol and air-dry fixed samples showed no obvious changes in proportions (83–85% and 81–84%, respectively) to the control samples (84–85%). In addition, depending on RNA degradation, the non-fixed sample showed a significant 3ʹ bias (Fig. 1e), while the fixed samples showed even coverage from 5ʹ to 3ʹ ends.

Overall, our results showed the RNA degradation would lead to non-reproducible data, which does not reflect the original transcriptome of the tissue. However, dehydration-based fixation of frozen tissue slices greatly improved the sensitivity and reproducibility of the transcriptome analysis. In particular, ethanol fixation showed a high degree of similarity with the control data. However, chemical tissue fixation, such as ethanol fixation, can cause degradation of fluorescent molecules and a resulting decrease of the fluorescence intensities^16^. Hence, ethanol fixation is inappropriate for samples that require fluorescent cell labelling for visualization of specific anatomical regions. By contrast, air-dry fixed samples are less susceptible to fading of fluorescent intensities and can be stored for a long time and be carried by keeping them in a dried condition. Therefore, we used these fixation methods for different purposes in accordance to the use after tissue fixation.

### Lysis and RNA purification from fixed tissue microdissection samples for RNA-seq analysis

**Figure 2:**
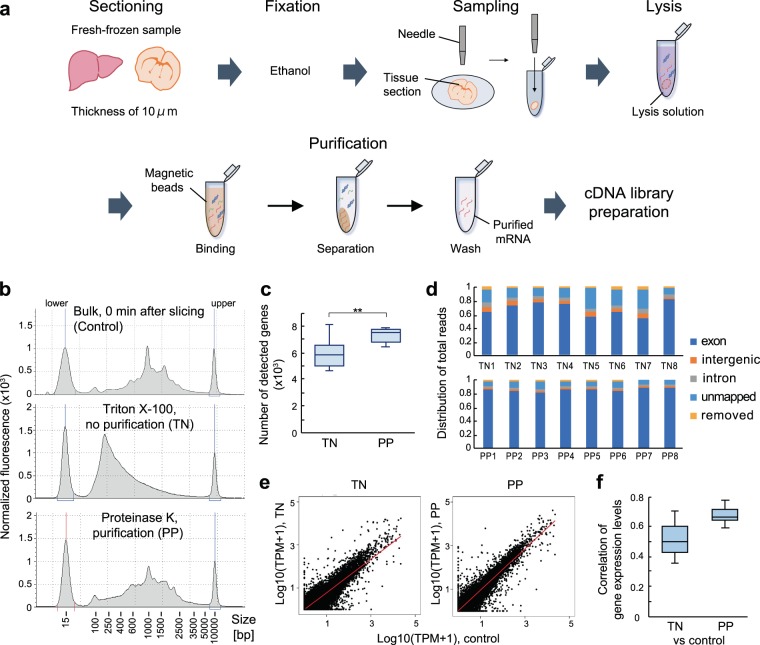
Evaluation of tissue lysis and RNA purification effects on RNA-seq from ethanol-fixed tissue microdissection samples. (**a**) Workflow of RNA-seq from tissue microdissection samples. The microdissection samples were collected from ethanol-fixed liver tissue using a punching needle. Then, the microdissection samples were lysed by Triton-X100 or Proteinase K, followed by poly(A) RNA purification by oligo (dT) magnetic beads. (TN: Triton-X100, no RNA purification and PP: Proteinase K and RNA purification) The tissue microdissection samples were serially collected from the same mouse liver slice. (**b**) Electropherograms of cDNA constructed under different tissue processing conditions. (**c**) The number of protein-coding genes estimated from RNA-seq results. Stars indicate p-value <0.005 determined by Welch’s t-test. (d) Sequencing read proportions assessed by mapping to a reference genome. (**e**) Comparisons of gene expression levels obtained between fresh tissue bulk RNA and tissue microdissection samples prepared under two different conditions. TPM values were averaged from four samples in the bulk sample pool and eight samples in the microdissection sample pool. (**f**) Pearson’s correlation coefficients across samples in the dataset including control samples and sample obtained by each method. Box plots show the within-sample range.

Since the tissue microdissection samples are small and contain a small number of cells, the standard column purification of RNA is not adequate for pre-processing toward RNA-seq library preparation. In order to recover a small amount of RNA from microdissection samples, we evaluated several tissue dissociation techniques combined with magnetic bead-based poly(A) RNA purification (Fig. 2a). In this study, we compared standard Triton X-100 lysis without bead purification (referred to hereafter as TN) and proteinase lysis with bead purification (referred to hereafter as PP). In Smart-seq2, cDNA synthesized from undegraded RNA mainly distributes around 500–2000 bp (Fig. 2b). However, TN showed short fragmented cDNA due to RNA degradation. The Proteinase K lysed and poly(A) RNA purification (PP) showed an improvement of cDNA length and comparable cDNA size proportions with bulk extracted RNA (Fig. 2b).

Next, we compared the qualities of RNA sequencing data from liver tissue microdissection samples treated with different RNA purification conditions. Although the cDNA was shortly fragmented, TN showed a certain number of detected genes (5932 ± 1122). As seen in the PP sample, Poly(A) RNA purification increased the number of detected genes (7329 ± 522, p < 0.005) and significantly decreased the coefficient of variation from 0.19 to 0.07 (Fig. 2c).

Moreover, we assessed the proportion of reads aligning to exonic regions, intronic regions, and intergenic regions (Fig. 2d). PP showed a great fraction of reads aligning to exons compared with others (86–90%). However, TN had lower proportions of exonic reads than bulk RNA extracted from the ethanol-fixed tissue (Figs. 1d, 2d). On the other hand, PP showed a highly reproducible proportion that is mainly derived from exonic reads. Thus, we considered that the PP condition could enrich the poly(A) RNA fraction from a small amount of biomolecules isolated from microdissection samples. Figure 2e,f show that PP showed a higher correlation of detected gene levels than TN (Pearson R = 0.68 ± 0.05 for PP and 0.51 ± 0.10 for TN). In addition, most genes in TN showed lower expression levels than the bulk sample. Significant lysis with Proteinase K could extract RNA efficiently so that it provides reproducible RNA-seq from tissue microdissection samples.

Overall, based on these results, we considered that Proteinase K lysis and magnetic bead-based poly(A) RNA purification is the most practical way to recover enough RNA from ethanol-fixed tissue microdissection samples. Based on the combination of tissue dehydration fixation, microdissection, efficient tissue dissociation, and RNA purification, we can analyse the tissue area-specific transcriptome in a reproducible and robust manner.

### Site-specific gene expression analysis of mouse brain tissue

**Figure 3:**
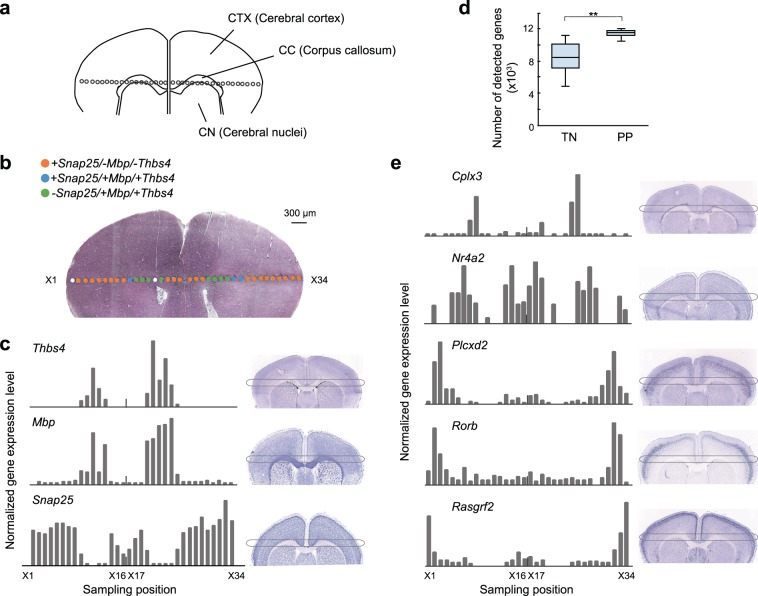
Site-specific gene expression determined from microdissection of the mouse brain. The microdissection samples were collected from ethanol-fixed mouse brain slices. Then, the microdissection samples were lysed by Proteinase K, followed by poly(A) RNA purification by oligo(dT) magnetic beads. Gene expression levels were determined from purified RNA with Smart-seq2. (**a**) Schematic image of collection points in the mouse brain. Microdissection samples were collected along CTX, CC, and CN. (**b**) Image of the mouse brain after collection of microdissection samples and Hematoxylin and eosin (HE) staining. All 34 points (X1 to X34) are indicated with different colours according to CTX, CC, and CN-specific gene expression patterns. Cutoff normalized TPM, *Thbs4*, 0; *Snap25*, 121; and *Mbp*, 1297. White means no data from microdissection. (**c**) Bar charts of normalized expression levels of CTX, CC, and CN-specific genes in individual microdissection points from X1 to X34. (**d**) The number of protein-coding genes estimated from RNA-seq results. The detected genes of TN were calculated from previously reported data^14^. Stars indicate p-value <0.005 determined by Welch’s t-test. (**e**) Bar charts of normalized expression levels of the prefrontal cortex layer-specific genes in individual microdissection points from X1 to X34. In (**c**) and (**e**), images on the right are *in situ* hybridization images acquired from the Allen Mouse Brain Atlas (http://mouse.brain-map.org/).

For showing the validity of the proposed method for site-specific gene expression analyses, we performed serial micro-dissectioning along a line crossing a frozen mouse tissue section fixed with ethanol (10 μm thickness, Fig. 3a,b). The obtained microdissection samples that were from areas including the cerebral nuclei (CN), cerebral cortex (CTX), and corpus callosum (CC), were lysed with Proteinase K, and the mRNA was purified using oligo(dT) magnetic beads. The cDNA libraries were prepared and analysed by Smart-seq2.

At first, we confirmed the expression level of the region specifically expressed at each site. We could classify the microdissection samples into three groups according to the area-specific gene expression patterns of *Thbs4, Mbp*, and *Snap5*. As shown in Fig. 3c, *Thbs4*, and *Mbp* were detected as highly expressed in CC among the microdissection samples X9-X13 and X20-X25. *Snap5* was detected as highly expressed in CTX among microdissection samples X1-X8, X14-X19, and X26-X34 (Fig. 3c). The distributions of these area-specific gene expressions were coincident with the observation of *in situ* hybridization images. Although we found inconsistent left-right symmetry in the gene expression patterns, it could be due to a misalignment of depth during tissue slicing.

In addition, the superficial layers of CTX are known to be classified into six layers^17^. We previously reported layer-specific expression of genes such as *Rasgrf2*, *Plcxd2*, and *Cplx3* in mouse brains using a microdissection punching system without tissue pretreatment and RNA purification technique^14^. In this research, 34 microdissection samples were collected along the left-to-right line of mouse brain and the symmetrical structure of the brain was confirmed by expression analysis with higher spatial resolution than the previous one. In addition, our method of tissue fixation and RNA purification increased the number of genes detected in each microdissection from 8598 ± 1669 to 11303 ± 771(p-value = 7.9e-13), and significantly decreased the coefficient of variation from 0.19 to 0.07 (Fig. 3d). The microdissection samples treated with PP showed a clear distribution of site-specific genes and gradual changes in expression areas along the microdissection points (*Rasgrf*2 for layer II/III, *Rorb* for layer IV^18^, *Plcxd2* for layers IV/V/VI, *Nr4a2* for layer VI^19^, and *Cplx*3 for layer VI) (Fig. 3e). As the microdissection samples were collected along the left-to-right line of sliced frozen tissue of the mouse brain, symmetrical layer-specific gene variation was detected.

This tissue processing technique would be combined other tissue collection systems such as LCM. In this study, we used Smart-seq2 for evaluation of RNA length integrity, but this pretreatment can be used for other RNA-seq protocols such as CEL-seq2 or other 3ʹ mRNA-seq^20–22^. Magnetic bead-based processing can be easily automated with an auto dispenser with magnetic stands. Hence, by combining our pretreatment and RNA-seq with the sample barcode and UMI sequence, we can process a large number of samples at one time. These technical combinations would be useful for understanding the cellular heterogeneity and spatial cell distribution in brain and cancer tissues.

Here, based on our microdissection punching system, we report a microtissue RNA-seq method with suppressing RNA degradation of fresh frozen tissue specimens by ethanol or air-dried fixation. By adding an efficient RNA purification step in the library preparation procedure, including tissue lysis with Proteinase K and mRNA capturing by oligo(dT) magnetic beads, we showed reproducible RNA-seq analysis from mouse brain and liver microdissection samples. The provided results on spatial transcriptomics analysis indicated that our microtissue RNA-seq procedure could be applied for analysing valuable samples such as pathological tissues.

## Conclusion

We developed a microtissue RNA-seq method while suppressing RNA degradation of fresh-frozen tissue specimens by dehydration fixation. This fixation method showed significant RNA stability in liver and brain tissue slices and enough mRNA recovery rate for RNA-seq analysis. It resulted in reproducible sequence read mapping in the exon region and detected more than 2000 genes compared to non-treated samples in the RNA-seq analysis. RNA-seq of serial microdissection samples in the mouse brain showed clear spatial gene distributions in a more reproducible manner than that observed in our previous study. Our method would be applied to various transcriptome analysis, providing information for region specific gene expression in tissue specimens.

## Methods

### Preparation of sliced frozen tissues from mice

Sliced frozen tissues were prepared as described previously^14^. All mice (ICR, male,> 10 months old, Tokyo Laboratory Animals Science Co. Ltd., Tokyo, Japan) were treated according to the protocols approved by the Committee for Animal Experimentation of the School of Science and Engineering at Waseda University (No. 2017-A056, No. 2018-A067) and in accordance with the law (No. 105) passed by and notification (No. 6) of the Japanese Government. The mice were euthanised, and the brain, liver, and kidney were immediately isolated. Each mouse tissue was embedded in SCEM solution (Leica Microsystems, Tokyo, Japan), rapidly frozen in liquid nitrogen, and stored at −80 °C prior to cryosectioning. The embedded surface was trimmed with a cryo-microtome (Leica) and then cryosectioned at a thickness of 10 μm. For preparation of ethanol-fixed tissue samples, the tissue sections were transferred on a cryo-film (SECTION-LAB, Hiroshima, Japan) and then soaked in 99.5% ethanol for 10 s. For preparation of air-dry fixed tissue samples, the tissue sections were transferred onto a PPS flame slide (Leica) and dried up by 5-min air blowing with a handy circulator. For the evaluation of RNA degradation, each sample was kept at room temperature. For preparing control samples, tissue sections were lysed 30 min after slicing by the addition of 700 μl of RLT buffer (QIAGEN, Tokyo, Japan) with 1/100 beta-mercaptoethanol (Wako Pure Chem Co., Osaka, Japan) added according to the manufacturer’s instructions.

Whole images of tissue sections were captured by microscopic imaging device (BZ-X710, KEYENCE) before and after collection of microdissection samples with tissue punching system. According to the tiled images of tissue, we had assigned the anatomical areas of interest and collated with gene expression data which have been individually indexed in RNA-seq library.

### Collection of microtissue using the semi-automated tissue microdissection punching system

The tissue microdissection punching system was fabricated by Frontier Biosystems (Tokyo, Japan). The hollow punching needle was made of stainless steel purchased from Castec (Kanagawa, Japan). It had a knife-edge of 5 μm diameter or less to cut tissue sections smoothly. It was connected to an injector filled with a buffer solution by a polytetrafluoroethylene tube (Nichias, Tokyo, Japan). Before capturing tissue microdissection samples, the inside of the hollow punching needle was pre-washed with 70% ethanol, RNase Zap (Thermo Fisher Scientific, MA, USA), and 99.5% ethanol (Wako). The sample table was equipped with a dish approximately 35 mm in diameter. We coated its inner surface with a silicon sheet (Kenis, Osaka, Japan) of 0.05 mm thickness. Occasionally, double layers of a silicon sheet were generated to capture a microdissection together with a part of the film. The collection position was manually selected while observing the tissue with a microscope, and the punching operation was carried out continuously.

### Total RNA extraction from tissue section

Total RNA was extracted from fresh-frozen mouse tissues before and after fixation using the RNeasy mini kit (QIAGEN) according to the manufacturer’s protocol and stored at −80 °C. The RNA integrity number equivalent (RINe) was measured by the Tapestation 4200 (Agilent, Tokyo, Japan). RNA concentration was measured using Qubit (Thermo Fisher).

### Preparation of the cDNA library from total RNA and microtissues

For the analysis of purified RNA, 50 or 500 pg of total RNA extracted from each tissue section was used as template for cDNA synthesis. The sizes and amount of cDNA were measured by Tapestation 4200. The concentration of cDNA brought to the sequencing library preparation were measured by Qubit ds DNA HS Assay Kit.

For the analysis of direct RNA-seq library preparation from microtissue samples, we used two different approaches; (1) tissue lysis with 0.1% Triton X-100, referred to hereafter as TN, and (2) tissue lysis with 1.2 μg/μl Proteinase K followed by poly(A) RNA purification using oligo(dT) magnetic beads, referred to hereafter as PP. For tissue lysis in the TN samples, 5.3 μl of cell lysis mixture (0.1% Triton X-100, 1 U/μl RNase inhibitor) was added and incubated at 72 °C for 3 min. For tissue lysis in the PP samples, 5.3 μl of cell lysis mixture (PKD buffer (QIAGEN): Proteinase K (QIAGEN) = 16:1) was added and incubated at 56 °C for 1 h. For the mRNA purification, the oligo dT magnetic beads (Dynabeads Oligo (dT)25, 61002, Thermo Fisher) were washed with the same volume of 1× hybridization buffer (2× SSPE, 0.05% Tween20, 0.0025% RNase inhibitor) three times and then were suspended in half volume of 2× hybridization buffer according to instruction manual. Washed magnetic beads were mixed with each tissue lysate. The bead-mixed tissue lysates were heated at 56 °C for 1 min and then were incubated at 25 °C for 10 min for annealing poly(A) RNA with Oligo (dT). The samples were subsequently incubated on a magnet stand for 2 min in order to collect magnetic beads. Then, the beads were washed with 100 μl of ice-cold 1× hybridization buffer twice, and then were washed with 100 μl of ice-cold 1× PBS with 0.0025% RNase inhibitor. The poly(A) RNA was then eluted into 2.8 μl of nuclease-free-water by heating at 80 °C for 2 min on a heat block, followed by magnetic separation on a magnetic stand and collection of the supernatant. The total amounts of lysed and purified samples were directly processed according to Smart-seq2 protocol^23^.

### Sequencing and data analysis

Sequencing and data analysis was carried out as described previously^14^. Amplified cDNA (0.25 ng) was used for preparation of the sequencing library using the Nextera XT DNA library prep kit. Paired-end sequencing was performed on the Miseq, with 75 bases for read 1 (R1) and 75 bases for read 2 (R2). We trimmed the adapter sequences in all the sequence reads using flexbar 3.3.0. The trimmed sequence reads were aligned to the Ensembl mouse reference genome (GRCm38 ver.92) for mouse brain tissue samples including the ERCC sequences using hisat 2 2.1.0 with the default parameters. The gene expression levels, given as transcript per million (TPM), were calculated using samtools 1.9 with a transcriptome reference obtained from Ensembl. The mitochondrial genes and ribosomal genes were removed from the TPM data because they fluctuated from sample to sample and gave high abundances.

### Accession number

The sequence data have been deposited in DNA Data Bank of Japan (DDBJ) under the accession number DRA008756.

## Supporting information

Supporting information.
