# Supplementary material for: Effective microtissue RNA extraction coupled with Smart-seq2 for reproducible and robust spatial transcriptome analysis
Source: Sci Rep. 2020 Apr 27;10:7083. doi: 10.1038/s41598-020-63495-6 (PMC7184581; doi:10.1038/s41598-020-63495-6)
Supplement: Supplementary file 1 — Supporting information. [file 41598_2020_63495_MOESM1_ESM.docx]

**Supplementary Information: Robust and reproducible RNA-seq from serial tissue microdissections for spatial transcriptomics**

Miki Yamazaki1,2†, Masahito Hosokawa3†, Koji Arikawa4, Kiyofumi Takahashi4, Chikako Sakanashi4, Takuya Yoda1, Hiroko Matsunaga4, Haruko Takeyama1,2,3,4*

^1^ Department of Life Science and Medical Bioscience, Waseda University

^2^ CBBD-OIL, AIST-Waseda University

^3^ Institute for Advanced Research of Biosystem Dynamics, Waseda Research Institute for Science and Engineering, Waseda University

^4^ Research organization for Nano and Life Innovation, Waseda University

*These authors contributed equally.

Correspondence and requests for materials should be addressed to H.T.(email: haruko-takeyama@waseda.jp)

**Supplementary Figure S1**. Evaluation of RNA degradation in tissue section of different organs. The mouse liver tissue, mouse kidney and mouse brain were incubated at 25 ℃ for 0 minutes or 30 minutes after slicing and then total RNA were extracted from each tissue. The tissues were serially sectioned from same mouse organs and used for RNA extraction. (a) Electropherograms of RNA extracted from different mouse tissues sections: liver, kidney brain. (b) The number of protein-cording genes estimated from RNA-seq. (c) Sequencing read proportions assessed by mapping to reference genome. (d) Normalized average read coverage shown across the percentile predicted transcript length (5′ to 3′). Protein cording genes were used to calculate the gene coverage.
